# A Comprehensive Evaluation of Disease Phenotype Networks for Gene Prioritization

**DOI:** 10.1371/journal.pone.0159457

**Published:** 2016-07-14

**Authors:** Jianhua Li, Xiaoyan Lin, Yueyang Teng, Shouliang Qi, Dayu Xiao, Jianying Zhang, Yan Kang

**Affiliations:** 1 Department of Biomedical Informatics, Sino-Dutch Biomedical and Information Engineering School, Northeastern University, Shenyang, Liaoning, China; 2 Key Laboratory of Medical Image Computing of Northeastern University, Ministry of Education, Shenyang, Liaoning, China; 3 Department of Biomedical Imaging, Sino-Dutch Biomedical and Information Engineering School, Northeastern University, Shenyang, Liaoning, China; 4 Border Biomedical Research Center, Department of Biological Sciences, The University of Texas at El Paso, El Paso, Texas, United States of America; NIDCR/NIH, UNITED STATES

## Abstract

Identification of disease-causing genes is a fundamental challenge for human health studies. The phenotypic similarity among diseases may reflect the interactions at the molecular level, and phenotype comparison can be used to predict disease candidate genes. Online Mendelian Inheritance in Man (OMIM) is a database of human genetic diseases and related genes that has become an authoritative source of disease phenotypes. However, disease phenotypes have been described by free text; thus, standardization of phenotypic descriptions is needed before diseases can be compared. Several disease phenotype networks have been established in OMIM using different standardization methods. Two of these networks are important for phenotypic similarity analysis: the first and most commonly used network (mimMiner) is standardized by medical subject heading, and the other network (resnikHPO) is the first to be standardized by human phenotype ontology. This paper comprehensively evaluates for the first time the accuracy of these two networks in gene prioritization based on protein–protein interactions using large-scale, leave-one-out cross-validation experiments. The results show that both networks can effectively prioritize disease-causing genes, and the approach that relates two diseases using a logistic function improves prioritization performance. Tanimoto, one of four methods for normalizing resnikHPO, generates a symmetric network and it performs similarly to mimMiner. Furthermore, an integration of these two networks outperforms either network alone in gene prioritization, indicating that these two disease networks are complementary.

## Introduction

The identification of disease-causing genes is important to researchers and clinicians for diagnosing and treating diseases. However, identifying these genes is time consuming and costly. A common practice is identifying and prioritizing candidate genes *in silico* and then validating these genes *in vivo*. Some diseases with overlapping phenotypes may be caused by different genes that belong to the same functional module. Many genes exert their functions as components of protein complexes. It has been observed that direct and indirect protein-protein interactions often lead to similar phenotypes.

Online Mendelian Inheritance in Man (OMIM) [1] is a comprehensive database for human genetic diseases and related genes that has become an authoritative source of disease phenotypes. Text mining of OMIM can be used to analyze human disease phenotypes. Computational approaches integrating phenotype similarity data with protein-protein interactions data have been developed to prioritize candidate genes [2–4]. However, OMIM is manually curated by disease experts, and phenotypic descriptions are found in free text. Therefore, standard vocabulary for phenotypic annotation is necessary for extracting clinical manifestations from OMIM records. Currently, three standard vocabularies are used for human phenotypic annotation in text mining of OMIM, i.e., Medical Subject Headings (MeSH) [5–7], Unified Medical Language System (UMLS) [8–12] and Human Phenotype Ontology (HPO) [13–15]. Similarity scores are calculated between disease phenotype pairs, and then disease phenotype networks (DPN) are created. Two DPNs based on MeSH and HPO are publicly available on the Internet. The former, called mimMiner [5], is the most widely-used for identifying disease genes, and the latter, called resnikHPO in this paper, is the first DPN annotated by HPO—a structured, comprehensive and well-defined set of terms dedicated to describing human phenotypic abnormalities [14].

The influence of resnikHPO is far less than that of mimMiner, mainly because resnikHPO is a text file that includes similarity scores between disease pairs from OMIM, Orphanet and DECIPHER, which is not convenient to use directly in programming. By contrast, mimMiner is a numerical matrix of similarity scores between human diseases from OMIM and is easy to use. However, OMIM has adopted HPO to annotate disease phenotypes; therefore, resnikHPO has potential applications in the future.

Which DPN is a better phenotype similarity network for prioritizing candidate genes? In this study, we aim to comprehensively compare the performance of mimMiner and resnikHPO on the same dataset, which, to our knowledge, has not been performed before.

## Materials and Methods

### Evaluation dataset

MimMiner was derived from the work of van Driel et al, who thought that the full text and clinical synopsis fields of OMIM records describe genetic disorders and used the anatomy and the disease sections of MeSH to automatically extract terms from the OMIM records. Thus, every record generated a feature vector. The similarity between the OMIM records was quantified by calculating the cosine of the angle between normalized vector pairs (Eq 1). By performing pairwise comparison of OMIM records, a disease phenotype network containing 5080 diseases was obtained, called mimMiner, whose arcs were weighted with a value in the range [0, 1]. mimMiner is a symmetric matrix in mathematics and can be found on http://www.cmbi.ru.nl/MimMiner/suppl.html.

$$Sim(A,B)=\frac{\sum_{i=1}^{l}A_{i}\times B_{i}}{\sqrt{\sum_{i=1}^{l}A_{i}{}^{2}}\times \sqrt{\sum_{i=1}^{l}B_{i}{}^{2}}}$$ (1)

ResnikHPO is based on HPO, which is constructed by using ontological concepts to represent clinical attributes of human diseases in the form of a directed acyclic graph. A pre-computed matrix containing pairwise similarity scores between 7477 human diseases was freely downloaded on September 1, 2014 from http://compbio.charite.de/hudson/job/hpo.diseasesimilarity/lastStableBuild/. The similarity between diseases pairs was calculated using Eq 2.

$$Sim(A\to B)=avg\left[\sum_{t_{1}\in A}\underset{t_{2}\in B}{\text{max}}IC(MICA(t_{1},t_{2}))\right]$$ (2)

Here, *MICA* is the most informative common ancestor of two terms *t*_1_ and *t*_2_ in ontologies. *IC* denotes the information content, which is defined as the negative natural logarithm of the frequency of a term used to annotate diseases.

*Sim*(*A*→*B*) is asymmetric with respect to A and B; therefore, the symmetric similarity measure is defined by

$$Sim_{symmetric}(A,B)=\frac{Sim(A\to B)+Sim(B\to A)}{2}$$ (3)

### Normalization and regression

The values ranges of *Sim*(*A*→*B*) and *Sim*_*symmetric*_(*A*, *B*) are not limited to [0, 1]. To compare resnikHPO with mimMiner, four methods were adopted to normalize the ranges, i.e., Lin, Sqrt, Maxmin and Tanimoto methods [16,17]. Let *Sim*(*A*, *B*) denote the similarity of disease phenotype sets A and B. *Sim*_min_ and *Sim*_max_ are the minimum and maximum value in a phenotype similarity matrix. The normalization is implemented using the following formulae:

Lin Method:
  $$Sim_{Lin}(A,B)=\frac{2\times Sim(A,B)}{Sim(A,A)+Sim(B,B)}$$ (4)
Sqrt Method:
  $$Sim_{sqrt}(A,B)=\frac{Sim(A,B)}{\sqrt{Sim(A,A)*Sim(B,B)}}$$ (5)
Maxmin Method:
  $$Sim_{\text{maxmin}}(A,B)=\frac{Sim(A,B)-Sim_{\text{min}}}{Sim_{\text{max}}-Sim_{\text{min}}}$$ (6)
Tanimoto Method:
  $$Sim_{Tanimoto}(A,B)=\frac{Sim(A,B)}{Sim(A,A)+Sim(B,B)-Sim(A,B)}$$ (7)

It should be noted that *Sim*(*A*,*B*) can be calculated using *Sim*(*A*→*B*), *Sim*(*B*→*A*) or *Sim*_*symmetric*_(*A*, *B*). Two suffixes -R and -C were used to distinguish row and column vectors of the asymmetric matrix of resnikHPO. The suffix -R corresponds to *Sim*(*A*→*B*), and -C corresponds to *Sim*(*B*→*A*). For example, after Lin normalization, three forms are available, i.e., Lin-R, Lin-C and Lin. The former two forms mean that the disease phenotype similarity data are extracted from the row and column vectors of the asymmetric matrix of resnikHPO, and the last form means that the disease phenotype similarity data are extracted from the symmetric matrix (i.e., *Sim*_*symmetric*_(*A*, *B*)).

As reported in an earlier study [2], logistic regression can strengthen the correlation between the similarity of two diseases and their causal genes. For a similarity value *x* of two diseases, the logistic function is defined by:

$$L\left(x\right)=\frac{1}{1+e^{\left(cx+d\right)}}$$ (8)

Where *d* = log (9999) and *c* is determined by leave-one-out cross-validation (LOOCV).

### Disease-gene validation

To access the performance of different DPNs, a large scale LOOCV experiments was adopted. Known associations between diseases and genes were extracted from the OMIM database. For each disease gene, the following experiment was conducted.

A gene was removed from the set of genes causing the disease. This removed gene was called the target gene, and the remaining genes in the set of genes composed the seed set. The target gene was predicted by prioritizing candidate genes. Because same gene or correlated genes could lead to overlapping disease phenotypes, protein—protein interactions (PPI) can be used to discover new genes [18]. All protein-coding genes in the PPI were also selected as candidate genes for prioritization, and the target gene was predicted according to the ranking.

For a polygenic disease, the seed set and phenotypic similarity data were used together to recover the association between the target gene and the disease. For a monogenic disease, there was no seed set after the unique gene was removed. As a result, the reconstruction of disease-gene relationship is mainly based on phenotypic similarity.

The disease-gene associations were extracted from the OMIM database, while the protein (gene) networks were obtained from Human Protein Reference Database (HPRD) [19], which contains 64662 unique interactions between 8919 proteins. According to a recent study [20], these datasets are available from http://genome2.ugr.es/prophnet/.

### Gene prioritization algorithm

Recently, a number of gene prioritization methods have been proposed, and the predictive performances were accessed. The network-based prioritization approaches, including Random Walk with Restarts (RWR) [21] and PRINCE [2], have shown more superior performances than the clustering and neighborhood methods [22]. Essentially, PRINCE is one type of Random-Walk based algorithm that has a slight advantage over RWR [2]. In this paper, the PRINCE algorithm was exploited to validate and predict disease genes.

PRINCE, as an iterative algorithm, can be defined as follows:

$$F^{t}=\alpha \times W_{norm}\times F^{t}{{}^{-}}^{1}+\left(1-\alpha \right)\times Y$$ (9)

where *W*_*norm*_ is a normalized form of the PPI network, *α* is a weight parameter and *F*^*1*^ = *Y*. In this study, the PPI consists of 8919 proteins. *Y* represents a prior knowledge function. If a protein is related to a considered disease, then *Y* assigns positive values to it and zero otherwise. Disease similarity information is incorporated into *Y* in proportion. The iteration continues until the difference between *F*^*t*^ and *F*^*t-1*^ falls below 10^−9^ or the maximum number of iterations (100) is reached.

### Evaluation criteria

To systematically compare the performance of different DPNs, the following evaluation criteria were used.

#### Mean rank ratio

The mean rank ratio (MRR) is defined as the average rank of the target genes among all candidate genes in LOOCV [20,23,24]. The MRR gives a comprehensive view of different DPNs for prioritizing candidate genes. A lower number for MRR indicates better performance.

#### Number of the top-ranking genes

In LOOCV, the proportion of true disease genes ranked at the top (with rank 1) is defined as the precision of prioritizing candidate genes [23,24]. A high precision means that the DPN has a high prediction power. However, the difference between different DPN forms is often minimal; therefore, the number of true disease genes ranked at the top is presented to accurately describe the prioritization performance.

#### True Positive Rate (TPR) in the top 5, 10 and 30 ranked genes

In a real situation, computer-aided gene screening usually gives a certain number of candidate genes. However, because validating disease-causing genes *in vivo* is expensive and time consuming, the number of candidate genes should be limited. The genes ranked within the top 5, 10 and 30 are sufficient for discovering the vast majority of genes; therefore, the TPRs in the top 5, 10 and 30 were selected as performance measures to estimate the efficiencies of the DPNs. These three thresholds represent reasonable biological hypotheses in a genetic screen [25], and they were adopted to evaluate gene prioritization tools or algorithms in previous studies [26]. The corresponding TPRs are the ratios of the number of true disease genes ranked in the top 5, 10 and 30 to the number of all validated disease-causing genes.

### Experimental procedure

The entire procedure consisted of the following four steps.

#### Step 1. Data extraction

The common disease records were extracted from the two DPNs to ensure that the diseases in the two datasets were consistent. There were 6485 OMIM disease records left in the resnikHPO after removing the Orphanet and DECIPHER-based diseases from all 7477 diseases. The mimMiner consisted of 5080 OMIM diseases. Consequently, 4366 diseases with same OMIM number were extracted from the two DPNs. For the resnikHPO, symmetric and asymmetric phenotypic similarity matrices with a size of 4366×4366 were created.

#### Step 2. Data normalization and regression

For the mimMiner, the extracted 4366×4366 matrix contained the values between 0 and 1. For the asymmetry and symmetry matrices extracted from resnikHPO, four methods, i.e., Lin, Maxmin, Sqrt and Tanimoto, were applied for normalization. Then, these data were processed with a logistic regression function.

#### Step 3. Validation and Evaluation

Together with a disease-genes network consisting of 4366 diseases and 8919 genes and a PPI network with a size of 8919×8919, the LOOCV was performed based on the PRINCE algorithm. In each run of LOOCV, the candidate genes were ranked, and the results were compared according to the evaluation criteria.

#### Step 4. Case studies

Two cases, i.e., OMIM 102200 and OMIM 114480 (Breast cancer), were provided to demonstrate the power of different DPNs in identifying new disease genes. The genes of OMIM 102200 were predicted without any available seed genes, and for the OMIM 114480, 18 new genes were prioritized with 4 seed genes.

**Fig 1:**
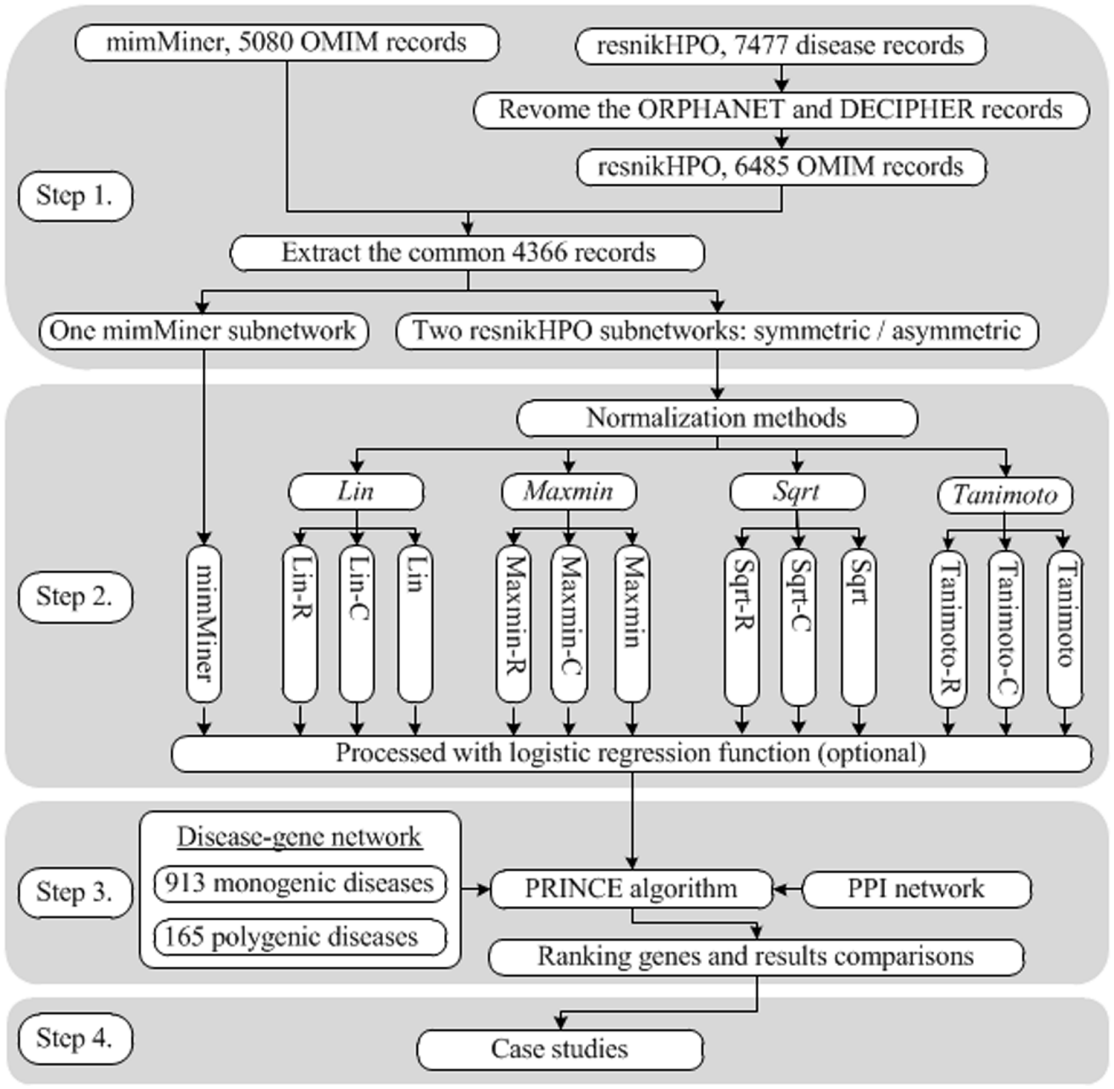
Flow-chart depicting the four steps of evaluating disease phenotype networks for gene prioritization. The first step consists of extracting the common disease records from mimMiner and resnikHPO to construct the corresponding subnetworks for comparative analysis. The resnikHPO has asymmetric and symmetric versions so that two subnetworks are extracted. Every subnetwork can be represented by an adjacency matrix. Step 2 consists of normalization and logistic regression. The asymmetric and symmetric subnetworks of resnikHPO need normalization to adjust all values into the range [0, 1]. Four methods shown in italics are adopted to process the corresponding adjacency matrix. Illustrated by the example of Lin method, the normalized asymmetric matrix is named Lin-R and Lin-C, while the normalized symmetric matrix is named Lin. In essence, Lin-R and Lin-C is the same matrix, but the difference is that the similarity scores between a given disease and the others are drawn from the row and column vectors of the matrix, respectively. In addition, mimMiner and resnikHPO can be integrated herein and the combination of mimMiner and Tanimoto outperforms each network alone (see the main text). Logistic regression is an optional process, but it substantially improves performance of the networks. Step 3 consists of validation and evaluation. Together with disease similarity as prior knowledge, 913 monogenetic diseases and 165 polygenetic diseases are input for gene prioritization in leave-one-out cross-validation (LOOCV) based on PRINCE algorithm using PPI network. Step 4 consists of case studies, involving gene prioritization for OMIM 102200 without seed genes and OMIM 114480 (Breast Cancer) with seed genes.

An overview of the evaluation procedure is presented in Fig 1.

## Results and Discussion

We first compared the performance of mimMiner and resnikHPO using a large-scale, LOOCV experiment and then evaluated the performance of their integration network. For a fair comparison, the shared 4366 diseases were extracted from the two disease phenotype networks. The 8919 protein-coding genes in the PPI network and the 4366 diseases formed a disease-gene network, containing 1340 associations between 1078 diseases and 897 genes. The 1078 diseases consist of 913 monogenic diseases and 165 polygenic diseases. Therefore, the LOOCV experiments were performed on monogenic and polygenic diseases. Then, different DPNs were statistically compared, and the *C* value in the logistic regression function was analyzed. Finally, two real examples demonstrate the ability of mimMiner, resnikHPO and their integration network for gene prioritization.

### Gene prioritization for monogenic diseases based on disease phenotype similarity

For each of the 913 diseases that were associated with only one gene, the disease-gene association was removed before the LOOCV. Then, the target gene was predicted using the previously known disease phenotype similarity information.

First, the original data, untreated by logistic regression, was validated. For the resnikHPO, each of the four transformations was validated individually, and each transformation included three different forms. The experimental results are listed in Table 1. The results show that for each transformation, the performance of the -C form was better than the corresponding -R and the symmetric form. Additionally, the symmetric form is a little better than the -R form because it is the average of these two asymmetric forms. It is notable that the Tanimoto-C correctly ranked 20 target genes at the top, and it is far superior to the other transformations.

**Table 1:** LOOCV results for 913 monogenic diseases, based on original disease phenotype networks.

Disease Phenotype Networks	MRR, %	Top-ranking genes^a^	TPR in the top 5, %	TPR in the top 10, %	TPR in the top 30, %
Lin-R	20.94	0	0.44	2.63	17.09
Lin	20.35	0	2.41	5.91	23.55
Lin-C	19.89	8	6.35	12.38	25.52
Sqrt-R	20.94	0	0.44	2.63	16.98
Sqrt	20.36	0	2.41	5.91	23.33
Sqrt-C	19.90	8	6.35	12.49	25.52
Maxmin-R	20.87	1	0.77	3.07	18.62
Maxmin	20.33	2	2.30	7.01	24.42
Maxmin-C	19.91	12	6.46	13.47	25.74
Tanimoto-R	20.66	4	2.74	6.35	23.00
Tanimoto	19.99	4	4.49	10.84	28.81
Tanimoto-C	**19.53**	**20**	**9.97**	**18.40**	**29.24**
mimMiner	20.96	1	3.40	6.24	20.92

MRR, mean rank ratio; TPR, true positive rate

^a^ It signifies the maximum number of target genes ranked on top of the candidate list. Totally, there are 913 target genes in LOOCV for monogenic diseases.

Second, the logistic regression was exploited to improve the performance of prioritization. Unexpectedly, the advantage of the -C forms disappeared and was even inferior to the -R forms. For example, Tanimoto-R restored 91 disease-gene associations among all 913 monogenetic diseases, while Tanimoto-C only recovered 73 disease-gene associations. In Table 2, these two Tanimoto forms are listed because they outperformed the others (for more details see S1 Table). The different values of *C* in logistic function were tested, and the best result based on the number of top-ranking genes was obtained when *C* was set to -13. Further studies showed that the symmetric forms of the DPNs were better than all asymmetric forms. As listed in Table 3, the symmetric forms of Tanimoto and mimMiner were better than the other three, especially when ranking the genes at the top and in the top 5. However, the overall performance of Tanimoto and mimMiner are quite close to each other. The parameter *C* varied with the distribution of DPNs and will be analyzed in the following section.

**Table 2:** LOOCV results for 913 monogenic diseases, based on asymmetric Tanimoto disease phenotype networks processed with the logistic regression function.

Disease Phenotype Networks	Best *C* value^a^	MRR, %	Top-ranking genes^b^	TPR in the top 5, %	TPR in the top 10, %	TPR in the top 30, %
Tanimoto-R	-13	19.95	91	20.04	25.96	35.16
Tanimoto-C	-13	19.00	73	19.17	26.62	35.60

MRR, mean rank ratio; TPR, true positive rate

^a^ the best *C* value will be obtained when maximum number of top-ranking genes is reached.

^b^ it signifies the maximum number of target genes ranked on top of the candidate list. Totally, there are 913 target genes in LOOCV for monogenic diseases.

**Table 3:** LOOCV results for 913 monogenic diseases, based on symmetric disease phenotype networks processed with the logistic regression function.

Disease Phenotype Networks	Best *C* value^a^	MRR, %	Top-ranking genes^b^	TPR in the top 5, %	TPR in the top 10, %	TPR in the top 30, %
Lin	-12	17.92	124	27.38	35.82	46.00
Sqrt	-13	17.81	120	27.60	35.16	46.22
Maxmin	-22	17.93	117	26.83	34.72	45.35
Tanimoto	-17	17.90	**134**	28.15	**35.93**	46.22
mimMiner	-14	**17.62**	133	**28.48**	35.38	**46.99**

MRR, mean rank ratio; TPR, true positive rate

^a^ the best *C* value will be obtained when maximum number of top-ranking genes is reached.

^b^ it signifies the maximum number of target genes ranked on top of the candidate list. Totally, there are 913 target genes in LOOCV for monogenic diseases.

### Gene prioritization for polygenic diseases based on seed genes and disease phenotype similarity

In the common 4366 OMIM records, there were 165 diseases with more than one associated gene. The number of genes associated with each of these diseases ranged from 2 to 5. A total of 427 disease-gene associations were extracted.

Unlike gene prioritization for monogenic diseases, the gene prioritization for polygenic diseases could take advantage of seed genes. In each run of LOOCV, the association of the disease-target gene was removed, and then using the known disease phenotype similarity, the remaining genes (namely seed genes) were used to identify the target gene. The performance differences of the three forms of resnikHPO are analogous to those in the LOOCV for monogenic diseases. For example, the -C forms were better than the corresponding -R and symmetric forms in prioritization performance when the original data were used, but the symmetric forms showed the best performance of the three when the similarity data were processed with the logistic regression function (for detailed data, see S2 and S3 Tables). The seed genes significantly improved the prioritization performance because of the so-called guilt-by-association principle. The validation results of the four symmetric forms of resnikHPO and mimMiner are listed in Table 4, in which the TPR in the top 5, top 10 and top 30 increases approximately 40% over their corresponding values in Table 3, while the MRR decreases approximately 50%. The overall performances of these five DPNs were similar to each other, but mimMiner performed slightly better than others. The remainder of this paper will focus on mimMiner and the symmetric forms of resnikHPO.

**Table 4:** LOOCV results for 165 polygenic diseases, based on seed genes and different disease phenotype networks processed with the logistic regression function.

Disease Phenotype Networks	Best *C* value^a^	MRR, %	Top-ranking genes^b^	TPR in the top 5, %	TPR in the top 10, %	TPR in the top 30, %
Lin	-12	**7.89**	80	39.34	47.78	62.06
Sqrt	-12	7.90	81	39.11	48.01	61.83
Maxmin	-18	7.96	81	36.77	46.37	61.12
Tanimoto	-18	7.91	**83**	38.88	48.48	61.59
mimMiner	-14	7.96	82	**42.39**	**50.59**	**64.17**

MRR, mean rank ratio; TPR, true positive rate

^a^ the best *C* value will be obtained when maximum number of top-ranking genes is reached.

^b^ it signifies the maximum number of target genes ranked on top of the candidate list. Totally, there are 427 target genes in LOOCV for polygenic diseases.

### Gene prioritization with integration of different disease phenotype networks

**Fig 2:**
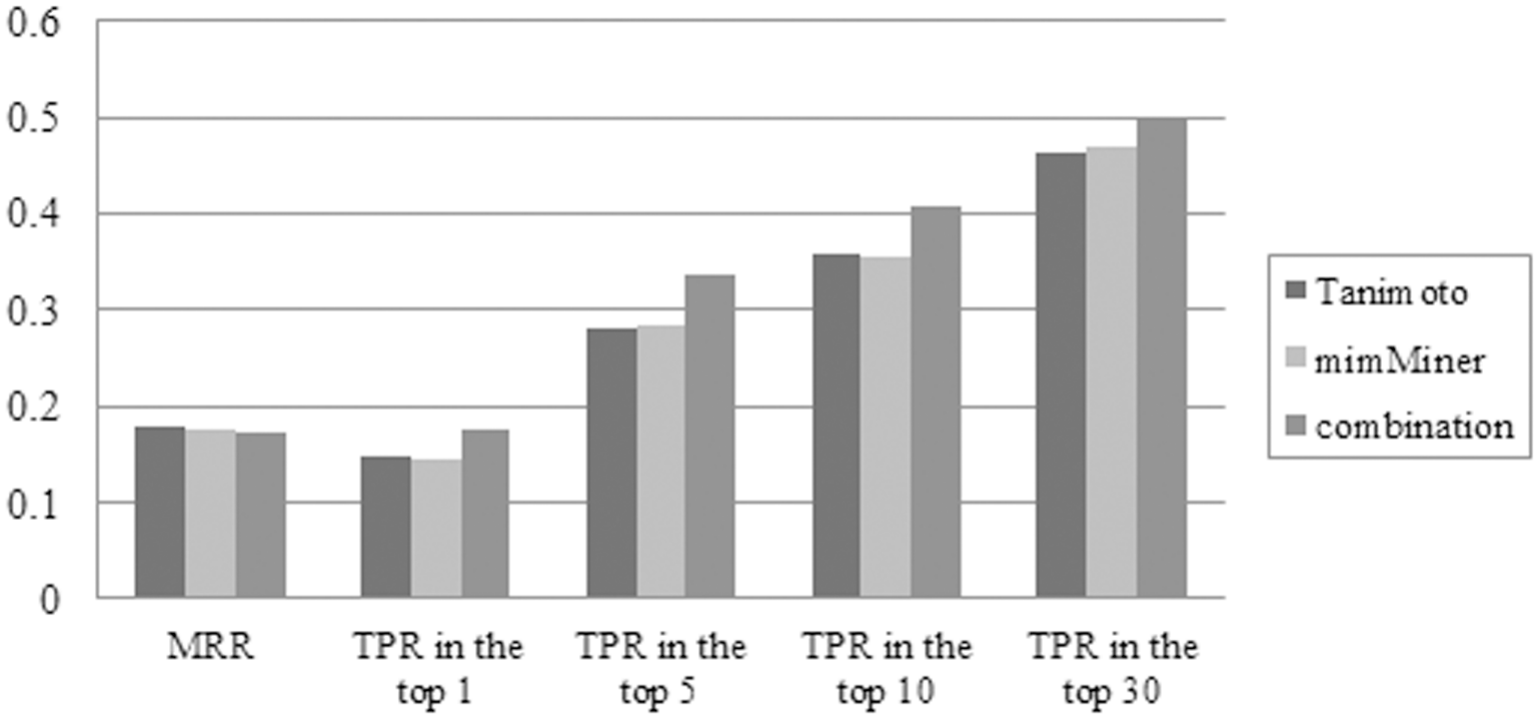
Comparison of the LOOCV best results for 913 monogenic diseases, based on Tanimoto, mimMiner and their combination. The prioritization performance of Tanimoto, mimMiner and their combination is compared based on the evaluation criteria including mean rank ratio (MRR), number of the top-ranking genes, and True Positive Rate (TPR) in the top 5, 10 and 30 ranked genes. For the number of top-ranking genes shown in this figure, its percentage in 913 genes (i.e. TPR in the top 1) was calculated.

The mimMiner and resnikHPO differ in vocabulary standardization and diseases similarity measures; therefore, it is possible that their integration could further improve the prioritization performance. Because its performance is the best of all normalized networks of resnikHPO, Tanimoto was selected to combine resnikHPO with mimMiner. Different component ratios affected the performance of the integrated network (Table 5). Clearly, the ratio of 0.5 led to the best result, which suggests that the combinational DPN is the arithmetic average of Tanimoto and mimMiner. In contrast to the best result of 134 genes in Table 3, the combinational DPN successfully identified 160 genes at the top with an increase of 19%. As shown in Fig 2, the combinational network has an obvious advantage over the individual Tanimoto or mimMiner.

**Table 5:** LOOCV results for 913 monogenic diseases, based on the combination of Tanimoto and mimMiner in different proportions.

Proportion of Tanimoto^a^	Best *C* value^b^	MRR, %	Top-ranking genes^c^	TPR in the top 5, %	TPR in the top 10, %	TPR in the top 30, %
0.3	-15	17.40	150	32.75	39.54	49.62
0.4	-16	17.27	155	33.52	40.42	49.95
0.5	-17	17.20	160	33.52	40.64	49.95
0.6	-17	17.29	159	33.52	40.85	49.18
0.7	-20	17.17	155	33.08	40.64	49.18

MRR, mean rank ratio; TPR, true positive rate

^a^ proportion of mimMiner = 1.0—proportion of Tanimoto.

^b^ the best *C* value will be obtained when maximum number of top-ranking genes is reached.

^c^ it signifies the maximum number of target genes ranked on top of the candidate list. Totally, there are 913 target genes in LOOCV for monogenic diseases.

**Fig 3:**
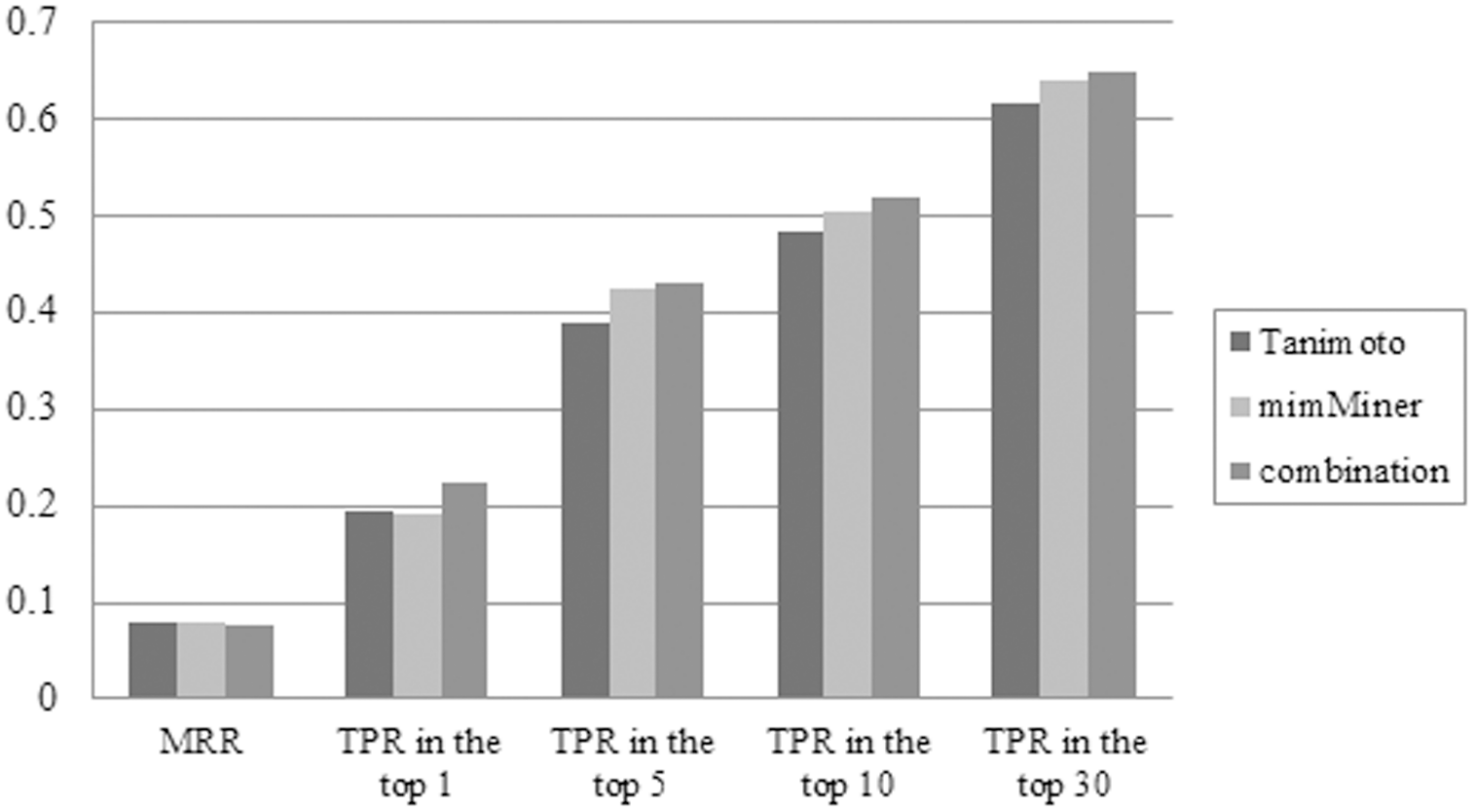
Comparison of the LOOCV best results for 165 polygenic diseases, based on Tanimoto, mimMiner and their combination. The prioritization performance of Tanimoto, mimMiner and their combination is compared based on the evaluation criteria including mean rank ratio (MRR), number of the top-ranking genes, and True Positive Rate (TPR) in the top 5, 10 and 30 ranked genes. Totally, there were 427 disease-gene pairs in the 165 polygenic diseases. For the number of top-ranking genes shown in this figure, its percentage in 427 genes (i.e. TPR in the top 1) was calculated.

The results of gene prioritization for polygenic diseases are listed in Table 6. The maximum number of top-ranking genes was 96 with the combinational ratio of 0.5. In contrast to the results of Tamimoto and mimMiner in Table 4, the corresponding MRR, numbers of top-ranking genes and all TPR were better in Table 6. As a whole, the MRR decreased 2 ~ 3% in the list of 8919 candidate genes, the number of genes at the top increased 16%, and the TPR in the top 5, 10 and 30 rose 11%, 10% and 3%, respectively. As shown in Fig 3, the combinational network was superior to the individual Tanimoto or mimMiner, especially for the numbers of top-ranking genes.

**Table 6:** LOOCV results for 165 polygenic disease, based on the combination of Tanimoto and mimMiner in different proportions.

Proportion of Tanimoto^a^	Best *C* value^b^	MRR, %	Top-ranking genes^c^	TPR in the top 5, %	TPR in the top 10, %	TPR in the top 30, %
0.3	-17	7.78	93	45.20	54.80	66.74
0.4	-17	7.76	94	44.50	54.80	66.28
0.5	-16	7.75	96	43.09	51.99	64.87
0.6	-16	7.75	94	42.62	51.99	64.40
0.7	-16	7.75	93	41.22	51.05	65.34

MRR, mean rank ratio; TPR, true positive rate

^a^ proportion of mimMiner = 1.0—proportion of Tanimoto.

^b^ the best *C* value will be obtained when maximum number of top-ranking genes is reached.

^c^ it signifies the maximum number of target genes ranked on top of the candidate list. Totally, there are 427 target genes in LOOCV for polygenic diseases.

Furthermore, the comparison of the mimMiner and Tanimoto with their combination was analyzed and listed in Table 7. For the monogenic diseases, there were 736 genes among all 913 genes that were not identified by mimMiner and the combination. The mimMiner successfully ranked 133 genes at the top of candidate list, including 116 genes that were also identified by the combination and 17 genes were only identified by mimMiner. When the combination was used, 44 top-ranking genes were identified only by the combination and 160 genes were totally ranked at the top. For the polygenic diseases, 7 genes were identified only by mimMiner, and 21 genes were identified only by the combination. As a result, the ratios of the gene numbers between the combination and mimMiner are 44/17 and 21/7, respectively. Similarly, as listed in Table 8, the ratios of the gene numbers between the combination and Tanimoto are 51/25 and 20/7, respectively. A common conclusion can be drawn: for monogenic diseases, the combination can correctly identify target genes approximately twice as well as Tanimoto or mimMiner alone, and for polygenic diseases, the corresponding ratio is approximately threefold. Undoubtedly, the performance of the combination is superior to those of mimMiner or Tanimoto alone.

**Table 7:** Comparison of the mimMiner with the combination of mimMiner and Tanimoto in LOOCV.

Validated by mimMiner or not (Y/N) ^a^	Validated by combination or not (Y/N) ^a^	913 genes of monogenic diseases	427 genes of 165 polygenic diseases
N	N	736	324
Y	Y	116	75
Y	N	17	7
N	Y	44	21

Y, yes; N, not

^a^ the target gene is successfully validated if it is ranked on top of the candidate list.

**Table 8:** Comparison of Tanimoto with the combination of mimMiner and Tanimoto in LOOCV.

Validated by Tanimoto or not (Y/N) ^a^	Validated by combination or not (Y/N) ^a^	913 genes of monogenic diseases	427 genes of 165 polygenic diseases
N	N	728	324
Y	Y	109	76
Y	N	25	7
N	Y	51	20

Y, yes; N, not

^a^ the target gene is successfully validated if it is ranked on top of the candidate list.

### Parameter sensitivity

The PRINCE (Eq 9) is not sensitive to parameter *α* when *α* is above 0.5 [2]; therefore, *α* was set to 0.9 in this study. However, the logistic regression function is crucial in the PRINCE algorithm; especially, the parameter *C* has a significant effect on the performance of the prioritization of candidate genes for a disease of interest. To comprehensively evaluate the performance, the maximum number of top-ranking genes in LOOCV for all 1340 disease-gene associations was selected as the evaluation criteria. The algorithm was tested on different values of parameter *C*, and then the best results for different DPNs were obtained. The relation between the distribution of DPNs and parameter *C* was studied. The total similarity of a disease with others was defined as the sum of its corresponding row in the matrix of DPNs. The ratio of total similarity of each disease in a DPN and its corresponding value in mimMiner was calculated. The ratios were summed and then averaged over all diseases, which was defined as the relative network mean. In general, the best parameter *C* and the relative network mean share the same changing trend in all six DPNs (Table 9).

**Table 9:** Best *C* values in different DPNs.

	Sqrt	Lin	mimMiner	Combination	Tanimoto	Maxmin
Relative Network Mean	1.3097	1.2694	1	0.8660	0.7319	0.6588
Best *C* value^a^	-12	-12	-14	-17	-17	-18
top-ranking genes^b^	201	204	215	254	215	196

^a^ the best *C* value will be obtained when maximum number of top-ranking genes is reached.

^b^ it signifies the maximum number of target genes ranked on top of the candidate list. Totally, there are 1340 target genes in LOOCV for monogenic and polygenic diseases.

**Fig 4:**
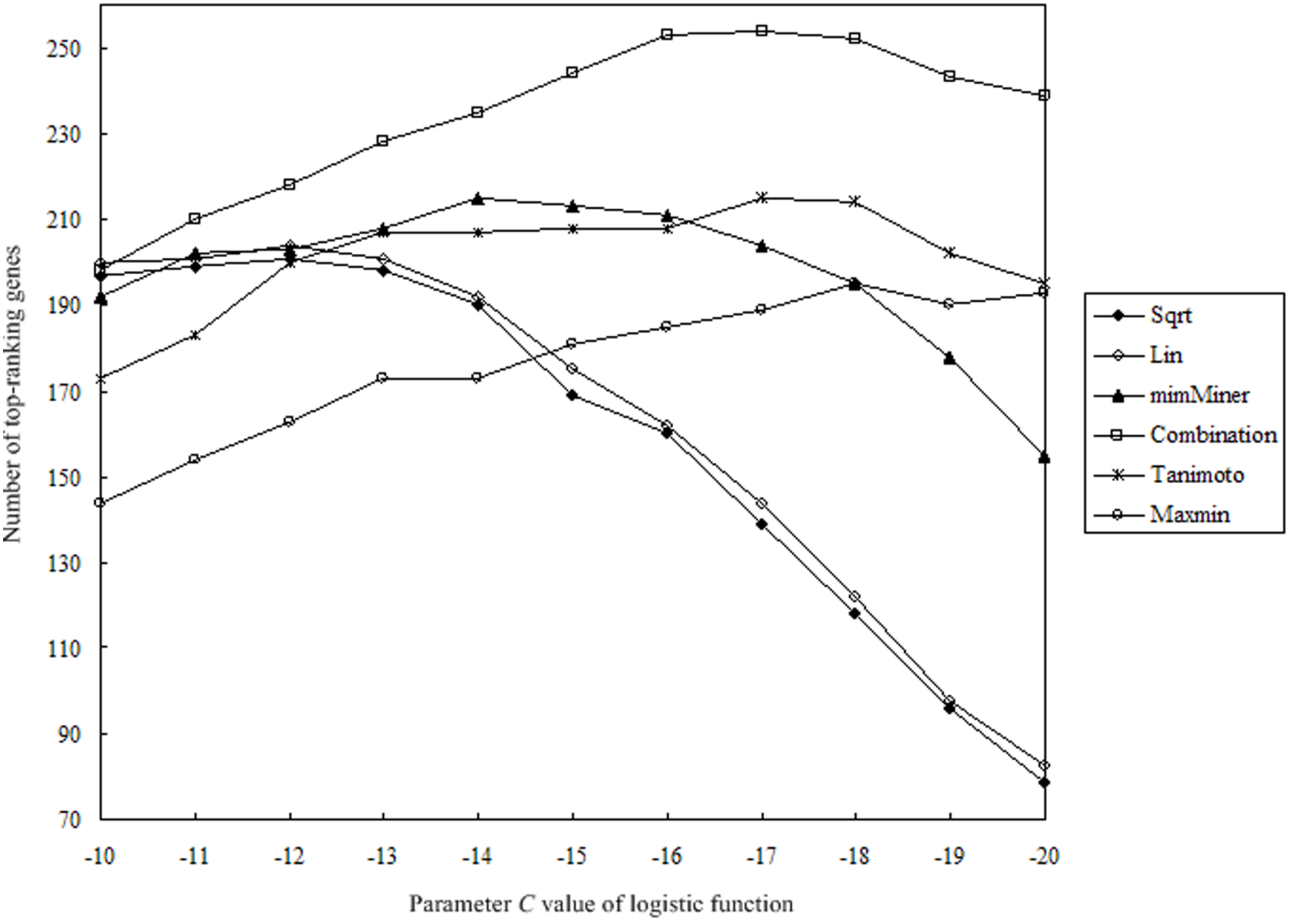
Correlation between parameter *C* of the logistic function and the number of top-ranking genes. Correlation curve of parameter *C* of the logistic function and the number of top-ranking genes is plotted, and the slow peak value is presented for each of the six disease phenotype networks. The combination is an integration of mimMiner and Tanimoto in the respective proportion of 50%.

Furthermore, different *C* parameters were tested with the steps 1 and 0.1. As shown in Fig 4, there is a slow peak value in performance on parameter *C*. With step 0.1, parameter *C* had a slight effect on the results (S4 and S5 Tables); therefore, it is reasonable that parameter *C* is assigned an integer value in practice. Additionally, it should be noted that the best parameter *C* was -15 in a previous report [2]. In fact, in contrast to the number 215 in Table 9, the total number of top-ranking genes was 213 when *C* was -15; thus, the difference was not very significant.

### Case studies

To evaluate the precision of different DPNs on real case studies, Tanimoto, mimMiner and their combination were used to predict the newly discovered genes in the OMIM database. In the experiment, the parameters were set to those that yielded better results. For the combined networks, the ratio of both Tanimoto and mimMiner was 0.5. Tanimoto, mimMiner and the combination were all symmetric networks that were processed with the logistic regression function with parameter *C* assigned -17, -14 and -17, respectively. The experiment included the prediction of new disease-gene associations with and without seed genes. In each case, the similarity scores of the query disease *q* with the others were obtained from mimMiner, Tonomoto or the combination. The prior knowledge *Y* (i.e., the possible relationship between *q* and each gene) was calculated based on the similarity scores and the disease-gene network. Using Eq 9, the prioritized genes of *q* were listed in *F* upon termination of the algorithm. The genes only in top 30 were considered.

For the gene predictions without seed genes, OMIM 102200 (pituitary adenoma, growth hormone-secreting, 1; PAGH1) was randomly selected from the diseases whose genes have all been recently discovered. There are two genes responsible for OMIM 102200, i.e., *GNAS* and *AIP*. The three DPNs were evaluated on how highly these genes were ranked. The results showed that all three DPNs ranked the two genes very lowly among 8919 genes. A method was adopted that only prioritizes all of the candidate genes contained in the linkage interval [14]. The candidate genes are the target gene and its neighborhood genes. In the experiment, 30 candidate genes were selected. However, some candidate genes were not included in the gene network (i.e., PPI network); therefore, only some of the candidate genes could be prioritized using the PRINCE algorithm. The results of gene prediction are presented in Table 10. As is listed in Table 10, the performance of mimMiner and that of the combination were similar, but Tanimoto exhibited the best performance because it yielded the highest ranks for the two new genes.

**Table 10:** Comparison of ranking for 2 genes of OMIM 102200, based on mimMiner, Tanimoto and their combination.

Target gene	Candidate genes in the linkage interval	Candidate genes in the gene network	MimMiner	Tanimoto	Combination
*GNAS*	30	15	1/15 ^a^	1/15	1/15
*AIP*	30	26	20/26	16/26	19/ 26

^a^ m/n signifies that the corresponding gene was ranked No. m in all n candidate genes.

For the gene prediction with seed genes, breast cancer (OMIM 114480) was studied. In the dataset of disease-gene associations used in this paper, breast cancer was related to 5 genes, i.e., *BRCA1*, *RAD51*, *BRCA2*, *NBN* and *PIK3CA*. However, the new dataset, which was downloaded from ftp://ftp.ncbi.nih.gov/gene/DATA/ on Jan. 19, 2015, showed that there were 24 genes in total responsible for breast cancer. Nevertheless, the *NBN* is no longer included; thus, there were 20 new genes. Furthermore, among these 20 genes, *SLC22A18* and *PALB2* were absent in the gene network used. As a result, in the prediction experiment, 4 genes of breast cancer were used as seed genes to predict the 18 newly discovered genes. All 8919 genes were prioritized using the PRINCE algorithm, and the genes in the top 30 are only worth testing further in the lab because of financial and time constraints. In other word, if any one of these 18 genes is ranked in top 30, it will be considered as a successfully predicted gene. For comparison, the best *C* (-14 for mimMiner, -18 for Tanimoto and -16 for the combination) obtained in LOOCV for polygenic diseases was also tested on each DPN. All three DPNs could rank 4 seed genes in the top 4 and 3 newly discovered genes *ATM*, *TP53* and *RAD54L* in the top 30 (S6 Table), but the combination always gave the lowest MRR of these three genes. The best result for each network was listed in Table 11. After the seed genes were removed from the list of prioritized genes, the MRR of the three genes for mimMiner, Tanimoto and the combination was 8.3, 9.3 and 6.7, respectively. Relatively, the combination reports a better result than the mimMiner or Tanimoto alone. As a whole, the performances of the three DPNs were similar in this experiment.

**Table 11:** Comparison of ranking for 22 breast cancer genes, based on mimMiner, Tanimoto and their combination.

No.	Gene	mimMiner^c^	Tanimoto ^c^	Combination ^c^
1	*RAD51*^a^	1	1	1
2	*BRCA2* ^a^	2	2	2
3	*BRCA1* ^a^	3	3	3
4	*PIK3CA* ^a^	4	4	4
5	***TP53***^b^	**13**	**5**	**5**
6	***RAD54L***^b^	**9**	**13**	**10**
7	***ATM***^b^	**15**	**22**	**17**
8	*BRIP1*	47	63	38
9	*CHEK2*	66	41	62
10	*ESR1*	72	73	70
11	*AKT1*	96	109	102
12	*BARD1*	128	64	108
13	*XRCC3*	199	271	205
14	*CASP8*	315	344	318
15	*PHB*	650	417	479
16	*TSG101*	657	476	511
17	*KRAS*	635	726	629
18	*CDH1*	688	701	674
19	*HMMR*	3661	3362	3337
20	*PPM1D*	5644	4328	4920
21	*RB1CC1*	5889	5619	5697
22	*NQO2*	6492	6168	6151

^a^ as seed genes.

^b^ three successfully predicted genes.

^c^ the *C* = -14 for mimMiner, -17 for Tanimoto and -16 for Combination.

## Conclusions

The purpose of this paper was to evaluate the DPNs and their application to gene prioritization. A comparative analysis of two classical DPNs, mimMiner and resnikHPO, was performed. The LOOCV was carried out to compare these two networks. Several conclusions can be made from the experimental results. The shared 4366 diseases, as a majority in the two DPNs, are analyzed and summarized in this study. Though the entire networks have not been evaluated because of incomplete data, the main research conclusions may be easily extended to the respective entire network.

First, the normalization of diseases phenotype similarity is often necessary when diseases are compared with each other, but different methods can lead to different results of gene prioritization. For the resnikHPO, all 12 normalization forms without logistic regression vary greatly on performance. For example, in Table 1 the TPR in the top 5 spans from 0.44% to 9.97%, and the difference is nearly 20-fold. The -C form of each asymmetric network (or matrix) yielded better results than the corresponding -R form and symmetric form. Among the 12 forms of resnikHPO, the performance of Tanimoto-C was the best in terms of the evaluation criteria. Tanimoto-C also performed better than mimMiner. For example, the ratio of the number of top-ranking genes is 20:1.

Second, logistic regression can significantly improve the performance of resnikHPO and mimMiner. In a sense, logistic regression suppresses the data noise in the DPN for gene prioritization. Under these circumstances, the symmetric form of resnikHPO has an advantage over the asymmetric form because of information fusion and noise suppression. Among of the four symmetric forms of resnikHPO, Tanimoto performed better than the others and performed similarly to mimMiner. To some extent, resnikHPO and mimMiner are complementary. The experimental results revealed that the best results were obtained when these two networks were integrated in the respective proportion of 50%. Relative network mean is proposed to describe the distribution of disease similarity relative to mimMiner. The optimal parameter *C* of logistic regression changes with respect to relative network mean and therefore parameter *C* can be roughly estimated when a new DPN is constructed.

Third, the development of a DPN based on the HPO is worthy of sustained attention, though there are no significant differences in gene prioritization between resnikHPO and mimMiner, the HPO is an ontological description of human phenotypic abnormality. The HPO has distinct advantages in the computational analysis of human phenotypes over MeSH terms and UMLS concepts when they are used in automated text-mining approaches. A main reason for this advantage is that the terms and structure of the HPO are manually curated according to medical knowledge. In addition, as far as resnikHPO is considered, the HPO contains 6485 OMIM diseases and 992 diseases from ORPHANET and DECIPHER databases, which is more than the 5080 OMIM diseases in mimMiner.

Finally, it is important to notice that gene prioritization tools failed to give high rank to causal genes for a lot of diseases whether a single or the combined DPN is used. The lack of necessary biomedical data, especially PPIs and disease-gene associations, may lead to low accuracy of prioritization. Phenotypically similar diseases often are related to common molecular mechanisms; therefore genes associated with similar diseases tend to encode proteins that are close in PPI networks [27,28]. Newly added proteins and interactions in PPI networks can change network topological features (i.e., betweenness centrality, closeness centrality, clustering coefficient and so on) and information propagation. As a result, the genes ranked low previously may be ranked near the top. In practice, multiply PPIs are integrated to improve the performance of prioritizing genes [29]. Computationally, the increase of number of similar diseases, accurate of diseases similarity and number of disease-gene associations can enhance the prior knowledge of specific disease, e.g. *Y* value in Eq 9. Thus, construction and analysis of human disease network have received sustained attention [30,31]. Unfortunately, research has shown that, by using only PPIs, essential genes in metabolic networks or transcriptional regulatory networks are unable to be predicted [32]. Obviously, the direction for future research in gene prioritization is to integrate different data sources and improve methodologies. The integration of various biomedical data (e.g., sequence similarity, functional annotation, microarray expression, protein domains, pathway membership and so on) and the combination of different methodologies (i.e., random walk, network flow and label propagation and so on) [28] should be more powerful and will lead to more accurate prediction [22]. However, in this study we aimed to estimate the usefulness of two DPNs. Therefore, the widely-used random-walk based algorithm and the PPI data were utilized for gene prioritization. Disease similarity analysis can also be used to predict the associations of disease-microRNAs [33,34] and calculate drug repositioning [35,36]; thus, the evaluation presented here and its applications have uses beyond gene prioritization.

## Supporting Information

S1 Table LOOCV results for 913 monogenic diseases based on the disease phenotype networks processed with the logistic regression function. (XLSX)

S2 Table LOOCV results for 165 polygenic diseases based on original disease phenotype networks. (XLSX)

S3 Table LOOCV results for 165 polygenic diseases based on the disease phenotype networks processed with the logistic regression function. (XLSX)

S4 Table LOOCV results for 165 polygenic diseases based on Tanimoto with different C values for the logistic regression function. (XLSX)

S5 Table LOOCV results for 165 polygenic diseases based on MimMiner with different C values for the logistic regression function. (XLSX)

S6 Table Comparison of the top 30 prioritized genes for breast cancer based on MimMiner, Tanimoto and Combination. (XLSX)
